# Pomegranate Extract Improves Maximal Performance of Trained Cyclists after an Exhausting Endurance Trial: A Randomised Controlled Trial

**DOI:** 10.3390/nu11040721

**Published:** 2019-03-28

**Authors:** Antonio Torregrosa-García, Vicente Ávila-Gandía, Antonio J. Luque-Rubia, María Salud Abellán-Ruiz, María Querol-Calderón, F. Javier López-Román

**Affiliations:** Department of Exercise Physiology, San Antonio Catholic University of Murcia (UCAM), 30107 Murcia, Spain; atorregosa@ucam.edu (A.T.-G.); ajluque@ucam.edu (A.J.L.-R.); msabellan@ucam.edu (M.S.A.-R.); mquerol@ucam.edu (M.Q.-C.); jlroman@ucam.edu (F.J.L.-R.)

**Keywords:** pomegranate, exercise performance, sports nutrition, delayed onset muscle soreness, muscle recovery, antioxidants

## Abstract

The efficacy of pomegranate (*Punica granatum*) extract (PE) for improving performance and post-exercise recovery in an active population was equivocal in previous studies. In this study, a randomised, double-blinded, placebo-controlled, balanced, cross-over trial with two arms was conducted. Eligibility criteria for participants were as follows: male, amateur cyclist, with a training routine of 2 to 4 sessions per week (at least one hour per session). The cyclists (*n* = 26) were divided into treatment (PE) and placebo (PLA) groups for a period of 15 days. After physical tests, the groups were exchanged after a 14-day washout period. Exercise tests consisted of endurance bouts (square-wave endurance exercise test followed by an incremental exercise test to exhaustion) and an eccentric exercise drill. The objective was to assess the efficacy of PE in performance outcomes and post-exercise muscular recovery and force restoration after a prolonged submaximal effort. Twenty-six participants were included for statistical analysis. There was a statistically significant difference in total time to exhaustion (TTE)(17.66–170.94 s, *p* < 0.02) and the time to reach ventilatory threshold 2 (VT2)(26.98–82.55 s, *p* < 0.001), with greater values for the PE compared to the PLA group. No significant results were obtained for force restoration in the isokinetic unilateral low limb test. PE, after a prolonged submaximal effort, may be effective in improving performance outcomes at maximal effort and might help to restore force in the damaged muscles.

## 1. Introduction

Polyphenols are natural occurring antioxidants and represent one of the most numerous and widely distributed groups of substances in the plant kingdom; as much as 8000 phenolic structures are currently categorized into four groups (flavonoids, stilbenes, lignans and phenolic acids) [1]. Polyphenols are widely found in foods including wine, green tea and red-coloured fruits such as pomegranate [2,3]. Some clinical intervention studies support the hypothesis of some cardiovascular benefits arising from polyphenol-rich beverages (red wine, tea and cocoa) [4,5]. In fact, epidemiological evidence suggests that polyphenols, at least in part, might explain the cardiovascular benefits from increased fruit and vegetable intake [6].

The pomegranate fruit includes bioactive substances such as hydrolysable tannins (gallotannins and ellagitannins), ellagic acid and its derivatives, gallic acid, anthocyanins/anthocyanidins, proanthocyanidins, flavonoids, vitamins, as well as sterols, lignans, saccharides, fatty acids, organic acids, terpenes and terpenoids, among others [7]. A study on different varieties of pomegranate showed that their total polyphenol content (TPC) and antioxidant activity vary depending on the part used and cultivar [8,9]; the TPC can be 20 times higher in the whole fruit than in arils [10]. Pomegranate juice is a source of polyphenols such as anthocyanins, flavanols and some ellagitannins (especially punicalagin), revealing a potent antioxidant activity that is three times higher than the well-known antioxidant properties of red wine or green tea [11].

The most abundant of these polyphenols in pomegranate juice is punicalagin [12], belonging to the ellagitannin subgroup (hydrolysable tannins), implicated as the bioactive constituent responsible for more than 50% of the juice’s potent antioxidant activity [13] but the content of polyphenols in commercial pomegranate juices varies according to variety and industrial manufacturing process with considerable variability in the punicalagin content [11]. It seems that most of the studies carried out with pomegranate extracts were juices with a high polyphenol content without a standardized punicallangin content.

Pomegranate juice has shown many beneficial effects on markers of cardiovascular health including: A)Blood pressure: Systolic blood pressure (SBP) and diastolic blood pressure (DBP) lowering, as showed in a meta-analysis (*n* = 322) expressed as the weighed mean difference (WMD) [SBP (WMD: −4.96 mmHg, 95% CI: −7.67 to −2.25, *p* < 0.001) and DBP (WMD: −2.01 mmHg, 95% CI: −3.71 to −0.31, *p* = 0.021)] [14]; and through decreased activity of related enzymes: serum angiotensin-converting enzyme (36% decrease) [15]; and possibly 11β-hydroxysteroid dehydrogenase type 1 enzyme activity [16]. B)Improved lipid metabolism: by a diminished low-density lipoproteins aggregation and an increased serum paraoxonase activity up to 20% [17] (an enzyme possessing atheroprotective properties [18]); and decreased glucose conversion to fat (by inhibition of basal glucose incorporation into lipids in human adipocytes [19]). C)Reduced markers of oxidative stress: by diminished lipid peroxidation in overweighed and obese humans after physical exercise [20] and against smoking (in rats), also showing increased levels of antioxidant enzymes [21].

In sport, polyphenols exert physiological effects that can increase by 1.90% [95% confidence interval (CI) 0.40–3.39] a diversity of athletic performance parameters – such as exercise time to fatigue, distance covered in a pre-selected time period, time to complete a certain distance and maximum power output – in both untrained but predominantly trained males [22]. Moreover, there are a number of both narrative and systematic reviews supporting the role of polyphenol supplementation in endurance performance [23,24,25,26] showing decreased rate of perceived exertion (RPE), increased maximal oxygen uptake and a faster recovery of muscle capacity (with a parallel trend to a faster decrease of inflammatory markers [27]).

Polyphenols have been purported to improve aerobic metabolism through stimulation of mitochondrial biogenesis (by increasing expression of genes encoding cytoprotective proteins [28] and activation of sirtuins [29] mediated by specific polyphenols such as catechins, resveratrol, quercetin and curcumin [30]). On the other hand, antioxidant supplementation may impair muscle performance by decreasing force production (by blocking oxygen delivery from blood to myocytes [31] and by modifying basal cellular redox state [32]) and training adaptations derived from physical stress [23,24,25,26].

The purpose of this study was (1) to test the hypothesis that pomegranate extract (PE), in dietary doses, can benefit endurance capacity (sub-maximal and maximal) after an extenuating bout and (2) the contribution of PE to post-exercise strength recovery after an exercise induced muscular damage.

## 2. Materials and Methods

### 2.1. Subjects

Thirty amateur endurance-trained male athletes (age: 34.9 ± 10.0 years; weight: 74.8 ± 11.3 kg; height: 1.75 ± 0.05 m; body mass index (BMI): 24.5 ± 3.0 kg/m^2^; maximal oxygen consumption (VO_2max_): 54.4 ± 9.0 mL/min/kg) volunteered to participate in the study. Inclusion criteria were as follows: (1) male aged between 18–55 years old; (2) amateur cyclist, with a training routine of 2 to 4 sessions per week, for at least one hour per session. Exclusion criteria were as follows: (1) allergy to pomegranate or any of its by-products; (2) serious clinical pathology or antecedents; (3) regular smoker; and (4) supplementation with ergogenic aids in the last 3 months. Participants were informed (verbally and written) of the purpose of this study, the characteristics of the product used for supplementation, its effects, as well as any possible risk and side effects resulting from the supplement and the procedures of the study. Subject were informed of their right to quit the study at any time, without the need to provide any reason. Participants gave written consent before the study was started. The study protocol and informed consent were approved by the Ethics Committee of the Catholic University of Murcia (UCAM) and were in agreement with the Declaration of Helsinki.

### 2.2. Trial Design

A double-blind, placebo-controlled, randomised, balanced, crossover design with two different study arms was used to test the effect of pomegranate extract (PE) or placebo (PLA) supplementation. Randomization was performed by a scientist not participating in the study, using software (Epidat 4.2, 2016) that generated random codes which were assigned to participants. An initial incremental exercise test to exhaustion (IETE) was carried out to make an initial assessment of the physical condition of each participant. Then, supplementation protocol of each group (PE or PLA) commenced (first allocation round), for a period of 15 days, after which cyclists underwent the exercise protocol (endurance and strength) to measure intervention effect. Afterwards, supplementation was discontinued 14 days for washout and the same procedure was repeated in the second allocation round (crossover design).

### 2.3. Supplementation Protocol

Participants ingested two capsules of PE (composition per capsule: 375 mg of POMANOX^®^ P30 with 30% punicalagins; total amount of punicalagins α + β per capsule: 112.5 mg) per day, immediately after breakfast; that is, a total dose of 225 mg punicalagins/day, for 15 days of treatment per study arm (or PLA (placebo): 15 days/15 days of experimental product), with 14 days of washout between them.

Both products, PE (POMANOX^®^ P30, EUROMED S.A., Barcelona, Spain) and PLA (maltodextrin) were identical in appearance: hard, orange-coloured capsules sealed in a 15-capsule aluminium blister pack, inside a cardboard box (3 blisters per box), properly labelled, randomized and identified. Storage instructions stated to store the product in a cool dry place, away from sunlight and intense odours.

Identification and quantification tests of the final product were performed by high-performance liquid chromatography (HPLC) according to the supplier reference standard, by a validated method of analysis (SOP No. HPLC-757). Compliance with the identification and quantification of the active ingredient, providing at least 30% of punicalagins α + β, was checked with the certificate of analysis provided by the manufacturer to ensure proper final product specifications.

#### 2.3.1. Compliance and Follow-Up

To ensure compliance and fulfilment, participants were given an extra blister pack provided as a backup (in case of accidental loss) and were asked to return empty blister packs and spare capsules after each intervention.

For the follow-up, participants were reminded verbally and through e-mail communication to consume the experimental supplements.

#### 2.3.2. Dietary Assessment and Control

The dietary habits of the participants were recorded using a validated food questionnaire. Subjects did not change their usual diet during the study period. On the day before and the same day as any performance test, volunteers had to comply with a previously detailed diet developed by a nutritionist, until the test was performed. This included refraining from taking caffeine and any other ergogenic aids or drugs that could affect performance measures. This measure was taken to ensure that the observations made were only due to the supplement and were not influenced by other modifications in the diet. Additionally, volunteers were asked for their meal intake when they arrived at the laboratory to check diet compliance. Any variation in diet was written in a control table by the nutritionist to keep track of diet modifications.

### 2.4. Exercise Tests

At every supplementation completion, a square-wave endurance exercise test (SWEET), followed by an IETE and a subsequent eccentric exercise drill were performed.

The purpose of the first two tests (endurance tests) was to assess performance outcomes. The purpose of the eccentric protocol was to evoke exercise induced muscle damage (EIMD) to assess the contribution of the supplement to post-exercise biomarkers; therefore, no performance data were collected for this drill.

All three tests were conducted sequentially in the same session after each allocation round, separated by a 29-day lapse (14 days for washout and 15 days in the other crossover supplementation arm). Athletes did not change their physical activity habits during the study and were told to avoid physical exercise the day before they performed the tests.

Environmental conditions (room temperature and humidity) were replicated in every exercise test for optimal conditions using room air conditioning system and were additionally measured during the tests.

#### 2.4.1. Initial Physical Assessment: Aerobic and Health Assessment

A preliminary IETE test was performed to assess the training status of volunteers at baseline, 7 days prior to commencement of the exercise tests. The purposes of this test were to (1) familiarize volunteers with the testing procedures and subjective feelings of the exercise tests; (2) determine submaximal external workload for each of the exercise tests (set at 60% and 70% of the VO_2max_); and (3) establish ventilatory thresholds of participants (corresponding the anaerobic ventilatory threshold to the ventilatory threshold 2 (VT2)).

Every participant used their own bicycle placed on the rear wheel, so repeatability was controlled by this corrective measures: 1)Front–rear slope-ratio was corrected to zero (using a front wheel riser) during the trial; 2)Bike configuration (gear set, saddle and handlebars) should be kept during the study; 3)Bike fitting (seat-post height and angle, handlebar reach, height and grip position) should be the same and; 4)Preferred pedalling system (use of cycling shoes and type of clip/cleat) should be consistent.

Test consisted of a 3-min warm-up at a self-paced intensity, followed by an IETE (initial load: 50 Watts (W), with a 35-W step increment every minute) on an electronically braked cycle ergometer (Cyclus2, RBM elektronik-automation GmbH, Leipzig, Germany) at a self-selected cadence between 60–100 revolutions per minute (RPM) on a fixed gear selected at the beginning of the test. Volunteers were verbally encouraged by the staff to exert maximal effort. Exhaustion was deemed to occur when the subject decided to stop, when pedal cadence dropped 20 RPM below the minimum cadence established (i.e., 40 RPM) or when power output could not be maintained.

Heart rate was monitored continuously using an electrocardiograph and oxygen consumption (VO_2_) was collected continuously during this test using an automated breath-by-breath system (Jaeger Oxycon Pro^TM^, CareFusion, Höchberg, Germany) calibrated before each test. All measures were analysed using software (LABManager 5.3.0.4, VIASYS Healthcare GmbH, Höchberg, Germany) and were stored in a personal computer for later recall. Maximal criteria were interpreted according to [33], defined as a plateau of VO_2_, respiratory quotient (RQ) above 1.10 and heart rate (HR) above 95% of the theoretical maximum HR.

Ventilatory aerobic and anaerobic threshold were plotted in a graph by using previously mentioned software and interpreted according to the three-phase model [34] by ventilatory equivalents (VE) [35]. VT2 was set as the intersection point between the carbon dioxide ventilatory equivalent (VE/VCO_2_) and the oxygen ventilatory equivalent (VE/VO_2_) against time - defined as the point in which pulmonary ventilation during exercise (VE) starts to increase at a faster rate than oxygen uptake (VO_2_). Time values to reach VT2 were provided by the same software when a vertical line was placed on this intersection point.

After completion of the initial IETE, subjects were familiarized with the eccentric drill and isokinetic test.

#### 2.4.2. Exercise Tests: Endurance Test and Strength Protocol

Once the supplementation protocol was completed, sets of different exercise tests were performed on the same day, which are summarised as follows:

1. Endurance test
  a. Square-wave endurance exercise test (SWEET), followed immediately by
  b. Incremental exercise test to exhaustion (IETE), followed by 5 min of rest.
2. Strength protocol
  c. Eccentric exercise drill.

##### Endurance Tests (SWEET and IETE)

a. Square-wave endurance exercise test (SWEET): A constant intensity cycling test was performed on the same electronically braked ergometer in same conditions. Subjects were instructed to complete a self-paced 10-min warm-up, without reaching initial load, followed by 90 min of SWEET with an individual load in watts, corresponding to 70% of VO_2max_ as calculated after a preliminary test. HR was continuously monitored using a pulsometer (Polar RS800CX, Polar Electro Oy, Finland) to double-check that athletes remained under VT2 at the given intensity, by screening the heart rate variability which showed significant correlation with VT2 in previous work [36]. To ensure proper performance, cyclists followed a hydration protocol, which was measured during the trial [37]. Subjects were asked to estimate their rate of perceived exertion (RPE) using the Borg scale [38] (scale from 1 to 20) after warm-up (10 min after commencement) and after 30, 50, 70 and 90 min (end of the test).
b. Incremental exercise test to exhaustion (IETE): Once the SWEET was completed, the maximal incremental cycling test was performed without interruption. Following 3 min of recovery at a self-selected intensity (never above the initial load), subjects performed a progressive incremental cycling test (initial load: 60% of VO_2max_) with the same equipment and conditions as the preliminary test. The difference now was that every step consisted of 3 min instead of one (i.e.,: 35-W increase every 3 min). Lactate samples were collected 1 min 40 s after completing the test by lancing the left ring-finger pad and were immediately analysed by a blood gas analyser (ABL90_FLEX_, Radiometer Medical APS, Copenhagen, Denmark). Subjects were then asked again to estimate their RPE.

##### Strength Protocol (Eccentric Drill)

**Scheme 1:**
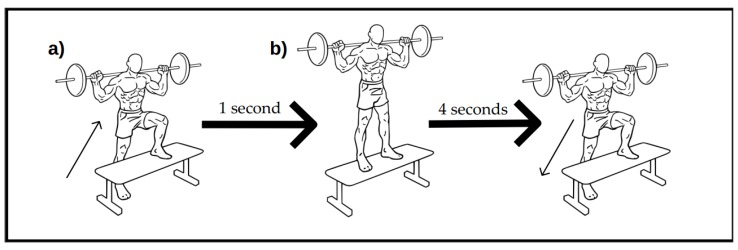
Eccentric exercise drill. The first picture (**a**) represents the initial position while (**b**) is the end position. The first sequence of the exercise consisted of moving from (**a**) to (**b**) and had to be performed in 1 s. Then, the initial position had to be recovered within 4 additional seconds. Both movements were considered to be 1 repetition. Pictures by © Everkinetic/http://everkinetic.com/, from the Wikimedia Commons project and distributed under a CC-BY-SA-3.0 license.

c. Subjects were given 5 min of transition time before performing the eccentric exercise test in another room with same conditions. The whole drill consisted of 15 repetitions, for a total of 6 sets per leg (15 × 6 = 90 repetitions per leg) performed at a specific cadence (1:4). The exercise sequence is depicted in Scheme 1.

A single-leg barbell step-up onto a bench, with no load and locked upper body (straight spine), was employed as described in Reference [39]. Subjects were given a plastic tube (200 cm long), which they had to place resting on their shoulders behind their head (with prone grip), forming a 45° angle between the forearm and arm. A fitness bench was placed in front of them, creating a 90° angle between the thigh and lower leg of the raised leg. Individual angles were measured by a goniometer prior to test, for bench height positioning. Subjects had to extend the leg placed on the bench, settling the other leg next to it (finishing the movement with both legs together and both knees extended) in 1 s, while reverting to initial position had to be completed in 4 s (consisting the full sequence of 5 s). Cadence compliance was facilitated by playing a free metronome app (Mobile Metronome 1.2.4F (2012), Gabriel Simoes, Google Play-Google LLC) at a speed of 60 beats per minute (bpm) in a 5/4 measure (with a different sound for the first beat of every measure) corresponding every beat to 1 s. One researcher supervised proper positioning and cadence of subjects during the drill.

### 2.5. Variables and Measurements

The following variables were measured: Initial physical assessment (IETE): Total time to exhaustion (TTE), oxygen consumption (VO_2_) and maximum oxygen consumption (VO_2max_). Endurance tests: Constant intensity endurance test (SWEET): Rate of perceived exertion (RPE). Maximal test (IETE): TTE, time to reach VT2 (time to VT2), VO_2max_, oxygen consumption at VT2 (VO_2_ at VT2) and lactate blood concentration (mmol/L) at the end of the test.

Post-exercise force recovery was measured by an isokinetic unilateral leg test (with the dominant limb) performed 2, 24, 48 and 72 h after the maximal IETE by a specially designed device (SYSTEM 3 PRO, Biodex Medical Systems Inc., Shirley, NY, USA) consisting of a dynamometer, chair and belts. Subjects had to perform maximal force output during a fixed angular velocity (60° per second) for both knee flexion and extension. The isokinetic variables measured were: Peak torque (N × m/Kg), relative work (J/Kg), work fatigue (%) and average power (W).

Evolution of muscular damage and inflammation biomarkers (creatine kinase (CK) and C-reactive protein (CRP), respectively) were measured by blood collection, 10 min before the first isokinetic test and subsequently upon each strength assessment completion (2, 24, 48 and 72 h later). Samples were divided and placed in vacuum tubes for either freezing or centrifugation and were immediately sent for laboratory analysis performed by an automatic analyser (IL Ilab 600, Chema diagnostica, Monsano AN, Italy).

**Scheme 2:**
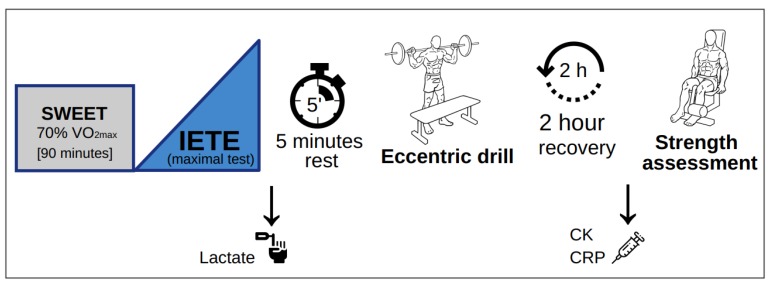
Experimental design. Acronyms are defined as follows; SWEET: square-wave endurance exercise test, VO_2max_: maximum oxygen consumption, IETE: incremental exercise test to exhaustion, CK: creatine kinase, CRP: C-reactive protein. Pictures by © Everkinetic/http://everkinetic.com/, from the Wikimedia Commons project and distributed under a CC-BY-SA-3.0 license. Icons made by Freepik from www.freepik.com and Pixelmeetup from www.flaticon.com.

A summary of the experimental design is depicted in Scheme 2.

### 2.6. Statistical Analysis

Quantitative variables are described as the mean, standard deviation and 95% confidence interval. This description was made for the total sample and was stratified by the randomized treatment arm. Qualitative variables are presented in tabular form, including the relative and absolute frequencies for the treatment groups and the global sample. Data were checked prior to analysis; in all cases, the Kolmogorov–Smirnov test was applied to test for a normal distribution and Levene’s test was used to test for homoscedasticity.

The evolution of these quantitative variables was analysed by parametric tests: a repeated measures *t*-test for the obtained variables from the IETE endurance exercise test and a two-way repeated measures ANOVA test with one within-subject factor (product) and one between-subject factor (time) for the variables obtained in the SWEET endurance exercise test and from the strength protocol. For the post hoc group comparison, the Bonferroni test was employed.

Statistical analysis was performed using SPSS software (version 21.0, Chicago, IL, USA) and *p* values are reported for every group and group × time interaction; *p* < 0.05 is considered statistically significant.

## 3. Results

### 3.1. Participant Flow Diagram and Baseline Characteristics

**Figure 1:**
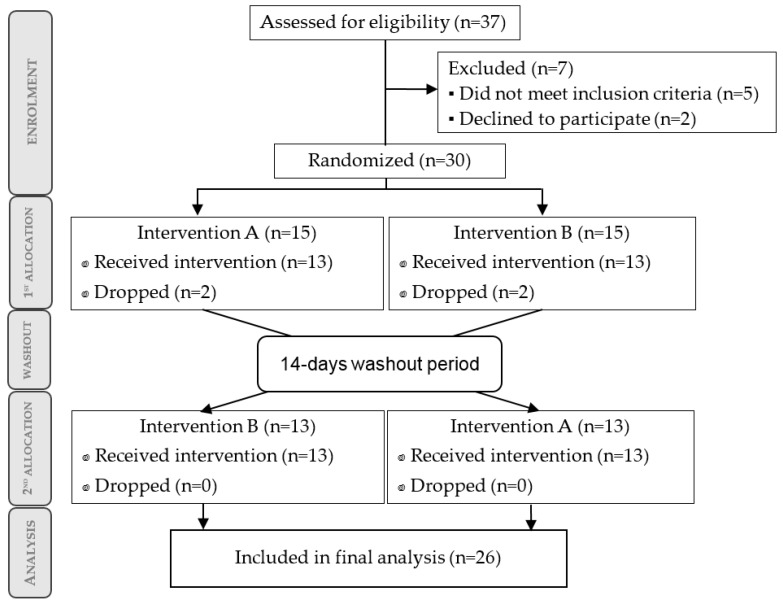
Participant flow diagram of the crossover design. Every group received both treatments (15 days of supplementation) separated by 14 days of washout.

The participant flow diagram is depicted in Figure 1.

A total of 37 subjects were initially recruited, of which seven were excluded due to non-compliance with the inclusion criteria (*n* = 5) or unwillingness to participate (*n* = 2). Thirty subjects met the initial screening criteria and were randomized. During the study, four of them (two from each arm) dropped out because of poor adherence to supplementation protocol. Finally, 26 subjects (13 subjects in each arm) received treatment successfully and were included for statistical analysis. All participants were cyclists competing at the regional level (Region of Murcia, Spain). The descriptive analysis at baseline is summarized in Table 1.

No significant differences were found for intra-subject values in any of the studied measures before each allocation. Therefore, we assumed that: (1) Participants started each intervention in same conditions; (2) Washout was successful in restoring values to baseline.

### 3.2. Conditions during Exercise Tests

No statistical difference was found for any of the measures in any group for temperature (*p* = 0.52), relative humidity (*p* = 0.97) or hydration (*p* = 0.77). During the PE tests, temperature was 22.12 ± 1.56 °C with a relative humidity of 57 ± 0.94%, whereas PLA temperature and relative humidity were 22.31 ± 1.57 °C and 57 ± 0.96%, respectively. Average water consumption was 1171.7 ± 304.8 mL for PE and 1156.3 ± 295.8 mL for PLA. Therefore, we assumed homogeneity of conditions among the performance trials for both groups.

### 3.3. RPE during and after the Square-Wave Test (SWEET)

The Borg’s scale results are presented in Table 2.

There was no statistically significant interaction effect between the effects of treatment and time on perceived effort. There was a statistically significant change in RPE for both groups from the beginning of the test (after the 10-min warm up), without a statistical inter-group difference. The average RPE reported for both groups was 13, corresponding in Borg’s scale to “somewhat hard”.

### 3.4. Incremental Exercise Test to Exhaustion (IETE)

IETE results are shown in Table 3.

There was a statistically significant difference in TTE and the time to reach VT2, with greater values for the PE compared to the PLA group. There was no statistically significant difference for VO_2max_, VO_2_ at VT2 or lactate.

### 3.5. Strength Protocol: Eccentric Exercise and Isokinetic Force

All subjects completed the eccentric exercise drill successfully. The isokinetic force results are shown in Table 4.

There was no statistical significance for the group × time values of any of the variables. There were greater values in PE for peak torque and relative work, both in extension and flexion values at 72 h. Work fatigue was lower for PE than for PLA, whereas average power was greater for PE in flexion and for PLA in extension.

### 3.6. Post-Exercise Muscular Damage and Inflammation

The evolution of muscular markers is presented in Table 5.

Baseline conditions were similar for both groups for CK and CRP. There was a significant change from baseline conditions for both groups for the time value but there was no statistical significance for CK and CRP between groups. Both CK and CRP were lower for PE than for PLA 72 h after the trial, especially CRP (27.96% lower than that observed in PLA).

## 4. Discussion

### 4.1. Primary Findings

The primary finding from this study was that 14 days of PE supplementation significantly increased the TTE of trained cyclists and the time to reach VT2 in a maximal test (IETE), after a long endurance effort (SWEET), compared to a placebo. This means that both maximal and submaximal performance increased. Previous studies of pomegranate supplementation on exercise performance and post-exercise recovery were limited and unclear, according to a recent meta-analysis [40]. To the best of our knowledge, this is the first study that has used a performance protocol combining an IETE after previous physical attrition in a SWEET to test the performance contribution of PE to performance outcomes. The physiological conditions of the physical protocol are similar to those occurring in long-distance competitions, when participants decide to increase their intensity in the last km/miles of the race, after a long submaximal effort and may be valuable when our aim is peak performance.

### 4.2. Acute Effect versus Chronic Benefits

Previous studies have observed performance improvements with an acute dosing of PE. Ellagitannin metabolites (including punicalagins) were previously studied in humans, showing a time to reach blood peak concentration of 1 h, with a clearance of 5 h for all participants [41,42]. In fact, ellagitannins are not absorbed as such; they are metabolized by the flora to yield up to 30 derived metabolites (mainly urolithins) [43]. In the current study, participants consumed the supplement during the morning, the same day of the tests, with a time lapse greater than 5 h from the test, so any possible acute ergogenic benefit from PE should be discarded due to an acute dose.

However, results in the current study are in line with a study on active runners, which found significant increases in TTE at 90% and 100% of peak velocity by a single dose of PE, 30 min before the trial, when performing a maximal test on a treadmill [44]. By contrast, a study with trained cyclists during high-intensity cycling in a high-altitude environment reported no overall significant effect from acute supplementation 2.5 h before the trial [45]. Our study was performed with a larger sample size (26 subjects instead of 8) and a chronic supplementation plan (15 days), which could have contributed to the shifting of the results. Furthermore, the current study seems to have the largest sample size of elite cyclists (*n* = 26) testing the supplementation of pomegranate for endurance performance and post-exercise recovery.

### 4.3. Pomegranate and Hypothetic Acute Effect through the Nitric oxide Pathway

Some of the acute effects of PE have been attributed to the nitrate content of pomegranate, which can have a role in nitric oxide (NO) formation during exercise. NO regulates physiological functions and has been recognized as an important factor in endothelial vascular relaxation during exercise [46,47,48] which also increases muscle blood flow [49]. Pomegranate significantly increase plasma nitrate (NO_3_) when supplemented for 8 days [50]. In addition, other polyphenol-rich supplements can produce a similar vasodilatation and contribution to NO pathways [51]. PE’s contribution to nitric oxide pathways could be due to its polyphenol content, which may protect the already produced nitric oxide against oxidative destruction, therefore enhancing its biological actions [52]. The current study administered PE for 15 days, which would be enough to elicit such benefits. However, as a scope limitation, nitrate concentration was not measured (neither in the product nor plasma as converted nitric oxide) since neither of these variables was within the scope of this study. Further research in this field is warranted for a better understanding of PE’s contribution to NO metabolic pathways.

### 4.4. Role of Pomegranate, Polyphenols and Punicalagins in Exercise Performance

To our knowledge, this is the first clinical trial that has studied the effect of a pomegranate extract standardized in punicalagins on exercise performance. The pomegranate extract used in this study is different in characteristics to other pomegranate extracts or juices regarding its polyphenol (and especially punicalagin) content. This ingredient (POMANOX^®^ P30) is made from whole fruits, only from Spanish cultivars and with dedicated technology and processing [53] being the only solvent used in the extraction step purified water, allowing the water-soluble polyphenols of the whole pomegranate fruit to be obtained.

Food supplements made of natural extracts can have a multifactorial effect, due to a rich variety of active substances (such as different types of polyphenols, depending on the source [54]), so specific mechanisms underlying their ergogenic effect are not easily deciphered. On the contrary, isolated active substances or standardized raw materials allow a more specific metabolic target but may hamper average supplement’s effect size by synergistic substances naturally present or by other unclear mechanisms.

As an example, isolated quercetin (a flavonoid from the flavonoid subclass of polyphenols) has been tested in sport performance without showing improvements [55,56] while the ellagitannin subclass of polyphenols, representing the majority of polyphenols in PE, has shown an increase in repeated sprint ability and running time to exhaustion, as well as blood flow and vessel diameter [44,57]. Isolated punicalagins in humans have been shown to have a particular pharmacokinetic effect – they are not easily converted to ellagic acid (another abundant ellagitannin molecule in pomegranate extracts) – which is indicative of behaviour different to other ellagitannin molecules [58]. Another example was found in a randomized trial with overweight patients (*n* = 49) assessing the contribution of PE to cardiovascular risk markers (18 in total), showing that this contribution may be inter-subject dependent or may respond to the baseline characteristics of subjects [59]. Thus, pomegranate extracts with standardized punicalagins, such as the extract used in this study or PE clustered with specific ellagitannin metabolites may behave differently. Future clinical trials on isolated active ingredients (such as punicalagins) would elucidate the specific metabolic and biochemical pathways taking place, which will be of great value for subsequent future research lines regarding exercise performance and health.

### 4.5. Strength Assessment and Force Restoration

PE was effective improving the recovery of force after an exercise induced muscle damage provoked by an eccentric exercise protocol in the upper body of recreationally active [60] and resistance-trained males [60]. However, this was equivocal for lower body isometric unilateral knee extension 2 h post-eccentric exercise [61] or 4 days after eccentric protocol [62], showing significant differences in the latter but not the former study. To our knowledge, the current study is the first to use an isokinetic (instead of isometric) unilateral strength protocol on lower limb muscles after an eccentric drill. The reason behind the non-significant results found in the current study could be due to sample size, which, for the previously mentioned study, reached significance with a population of *n* = 45 [62] but not in the one with *n* = 17 [61] or in the current study (*n* = 26). The only study found which employed an isokinetic test with pomegranate ellagitannins used a mix of ingredients in which these polyphenols represented a small portion of the beverage employed [63]. Thus, comparisons between the latter and the current study should not be made.

### 4.6. Post-Exercise Recovery

Previous work suggested that polyphenols prevent a rise in the muscular damage biomarker CK in a cycling test of same characteristics as the first endurance bout in the current study (SWEET, 90 min at 70% of VO_2max_) [64] which seems to have been effectively cancelled in the current study, by the eccentric drill design. A study found that pomegranate supplementation helped in restoring baseline values of both CK and CRP after weight lifting [65] which was not the case for trained cyclists after 17 days of supplementation with polyphenols [66]. In the current study, baseline values were almost restored after 72 h without conclusive results of PE’s contribution compared to PLA. Further research should be conducted to uncover more findings in this field.

## Figures and Tables

**Table 1:** Baseline demographic and clinical characteristics of participants. Values are presented as the mean with standard deviation (SD).

(*n* = 26)	Mean	SD
Weight (Kg)	74.8	11.3
Height (m)	1.75	0.05
Body mass index (Kg/m^2^)	24.5	3.0
Age (years)	34.9	10.0
Systolic blood pressure (mm Hg)	129.8	11.9
Diastolic blood pressure (mm Hg)	77.5	9.0
VO_2_ max (mL/min/Kg)	54.4	9.0
VO_2_ in VT2 ^1^ (mL/min/Kg)	41.6	8.5

^1^ Ventilatory threshold 2.

**Table 2:** Rate of perceived exertion (RPE) during the SWEET test. Borg scale (20-number scale), the lowest value corresponds to the lowest effort perceived and the highest to maximal perceived effort.

Group	RPE during SWEET (90 min)					Time *p*-Value	Group × Time *p*-Value
	after Warm-Up				End of Test		
	Min 10	Min 30	Min 50	Min 70	Min 90		
*PLA*	10.8 ± 2.6	11.9 ± 2.4	12.2 ± 2.3	12.7 ± 2.4	13.2 ± 2.3	< 0.001	0.74
*PE*	11.0 ± 2.2	12.1 ± 2.4	12.8 ± 2.0	13.2 ± 2.0	13.7 ± 2.3	< 0.001	

**Table 3:** Total time to exhaustion (TTE), time to reach ventilatory threshold 2 (time to VT2), maximal oxygen consumption (VO_2max_), oxygen consumption at ventilatory threshold 2 (VO_2_ at VT2) and lactate for the IETE. The difference is presented with a 95% confidence interval (CI).

	*PLA*	*PE*	Difference (95% CI)	Time *p*-Value
TTE (sec)	663.05 ± 222.70	757.35 ± 174.91	17.66–170.94	<0.02
Time to VT2 (sec)	533.33 ± 180.15	588.10 ± 154.75	26.98–82.55	<0.001
VO_2max_ (ml/min/Kg)	3957.75 ± 612.71	3871.35 ± 606.34	−257.27–84.47	0.30
VO_2_ at VT2 (ml/min/Kg)	3471.05 ± 473.87	3453.60 ± 500.27	−123.29–87.99	0.73
Lactate (mmol/L)	8.93 ± 2.43	9.23 ± 2.69	−0.73–1.32	0.55

**Table 4:** Isokinetic test values are presented as means with standard deviation. All units are presented in international units (IU); newton (N), meter (m), kilogram (Kg) and joule (J). For peak torque and work, relative values are given based on the subject’s total weight.

		Group	Post-Test				Time *p*-Value	Group × Time *p*-Value
			2 h	24 h	48 h	72 h		
**Extension (60°· s^−1^)**	**Peak Torque (N × m/Kg)**	*PLA*	250.8 ± 31.4	274.3 ± 37.2	276.6 ± 34.4	274.6 ± 38.6	<0.05	0.38
		*PE*	248.3 ± 43.2	267.1 ± 43.2	263.6 ± 63.0	279.4 ± 40.6	<0.01	
	**Relative work (J/Kg)**	*PLA*	188.5 ± 32.0	203.9 ± 31.6	200.0 ± 30.3	204.3 ± 32.4	<0.001	0.31
		*PE*	185.6 ± 32.0	200.7 ± 33.6	199.0 ± 52.1	215.8 ± 35.5	<0.001	
	**Work fatigue (%)**	*PLA*	14.3 ± 11.9	16.4 ± 10.5	16.6 ± 9.1	15.8 ± 8.1	0.81	0.38
		*PE*	9.8 ± 10.4	15.6 ± 11.0	14.6 ± 13.7	14.9 ± 13.0	<0.5	
	**Average power (W)**	*PLA*	120.7 ± 21.3	141.4 ± 22.8	144.8 ± 23.5	147.0 ± 26.9	<0.001	0.78
		*PE*	118.4 ± 32.8	134.2 ± 30.0	138.4 ± 39.5	146.6 ± 27.4	<0.001	
**Flexion (60°· s^−1^)**	**Peak Torque (N × m/Kg)**	*PLA*	132.7 ± 23.7	144.3 ± 28.3	147.7 ± 28.3	145.3 ± 28.1	<0.001	0.14
		*PE*	132.9 ± 33.1	134.3 ± 25.7	139.1 ± 38.1	148.9 ± 29.6	<0.01	
	**Relative work (J/Kg)**	*PLA*	101.6 ± 20.0	105.9 ± 27.1	104.1 ± 26.2	106.8 ± 22.7	0.34	0.28
		*PE*	99.4 ± 21.8	101.3 ± 20.1	103.1 ± 30.9	113.3 ± 22.2	<0.01	
	**Work fatigue (%)**	*PLA*	19.1 ± 11.5	22.0 ± 8.0	25.9 ± 8.8	23.1 ± 5.4	0.09	0.98
		*PE*	15.5 ± 18.2	16.3 ± 11.0	20.4 ± 9.4	18.9 ± 17.0	0.56	
	**Average power (W)**	*PLA*	66.9 ± 15.4	76.3 ± 16.9	77.2 ± 15.2	77.7 ± 17.4	<0.03	0.24
		*PE*	62.6 ± 18.5	68.3 ± 14.1	74.5 ± 22.0	79.7 ± 15.4	<0.001	

**Table 5:** Creatine kinase (CK) and C-reactive protein (CRP) values at baseline and 2, 24, 48 and 72 h after physical tests. Data were obtained using a paired two-tailed Student’s *t*-test. Data are presented as the mean with SD.

		Basal	Post-Test				Time *p*-Value	Group × Time *p*-Value
		0 h	2 h	24 h	48 h	72 h		
**CK**	*PLA*	119.00 ± 30.87	196.69 ± 126.51	313.94 ± 392.51	234.13 ± 215.20	184.88 ± 122.34	<0.05	0.71
	*PE*	114.63 ± 48.51	176.94 ± 101.18	254.06 ± 240.51	208.00 ± 153.81	169.81 ± 113.78	<0.02	
**CRP**	*PLA*	0.58 ± 0.34	0.57 ± 0.35	2.23 ± 2.12	1.59 ± 2.34	1.06 ± 1.40	<0.001	0.46
	*PE*	0.59 ± 0.27	0.60 ± 0.29	1.74 ± 1.08	1.06 ± 0.85	0.80 ± 0.56	<0.001	
